# Gut microbiome features and resistome elements associated with colonization and infection with antibiotic-resistance threats

**DOI:** 10.1080/29933935.2025.2570502

**Published:** 2025-10-26

**Authors:** Maliha Batool, Stephanie McMahon, Samantha Franklin, Corina Ramont, Pranoti Sahasrabhojane, Chia-Chi Chang, Tomo Hayase, Eiko Hayase, John C. Blazier, Robert Jenq, Samuel Shelburne, Jessica Galloway-Peña

**Affiliations:** a Department of Veterinary Pathobiology, College of Veterinary Medicine and Biomedical Sciences, Texas A&M University, College Station, TX, USA; b Interdisciplinary Graduate Program in Genetics and Genomics, Texas A&M University, College Station, TX, USA; c Department of Statistics, Texas A&M University, College Station, TX, USA; d Department of Infectious Disease, Infection Control and Employee Health, The University of Texas MD Anderson Cancer Center, Houston, TX, USA; e Department of Genomic Medicine, The University of Texas MD Anderson Cancer Center, Houston, TX, USA; f Texas A&M Institute for Genome Sciences & Society, Texas A&M University, College Station, TX, USA; g Department of Stem Cell Transplantation and Cellular Therapy, The University of Texas MD Anderson Cancer Center, Houston, TX, USA

**Keywords:** Shotgun metagenomics, gut microbiome, resistome, antimicrobial-resistant infections, colonization

## Abstract

Infection with antimicrobial-resistant (AR) pathogens is a leading cause of morbidity and mortality among patients with hematological malignancies; however, little is known about the gut microbiome dynamics in acute myeloid leukemia patients and its impact on AR infections (ARI) and/or colonization with AR pathogens (ARC). Longitudinal stool samples collected from 154 patients undergoing induction chemotherapy were analyzed using 16S rRNA sequencing, selective and differential media culturing, MALDI-TOF, and VITEK2 to identify patients with ARC or ARI and to isolate AR infectious and colonizing bacterial strains. Shotgun metagenomic sequencing of baseline stool samples revealed taxa abundances, resistome features, and KEGG pathways associated with AR-events. Baseline observed species were lower in patients with AR-events (*p* = 0.01). Although several baseline taxa were more abundant in AR-event patients, they were not statistically significant when they were corrected for false discovery. Functional analysis revealed that penicillin and cephalosporin biosynthesis pathways were significantly enriched in patients with ARC. In summary, identifying the baseline microbiome, resistome, and functional pathway biomarkers may forecast an increased risk of ARI and/or ARC, thereby informing antimicrobial treatment strategies in AML patients.

## Introduction

Infections caused by multidrug-resistant organisms MDRO are a significant public health concern. Understanding the factors contributing to antimicrobial resistance (AR) development is crucial for reducing infection rates and preserving the effectiveness of existing antimicrobial therapies.^1^^,^^2^ By 2050, AR pathogens are predicted to be the leading cause of death if no action is taken.^3^ The Center for Disease Control and Prevention (CDC) has categorized several microorganisms as “urgent”, “serious”, and “concerning” AR threats to global human health in the 2019 Antibiotic Resistance Threats report.^4^ This list included 5 urgent threats and 11 serious threats, including methicillin-resistant *Staphylococcus aureus* (MRSA)*,* multidrug-resistant *Pseudomonas aeruginosa* (MDRP), vancomycin-resistant *Enterococcus* (VRE), extended-spectrum beta-lactamase-producing Enterobacteriaceae (ESBL), and carbapenem-resistant Enterobacteriaceae (CRE).^4^

Published literature has demonstrated the critical role of the microbiome in providing gastrointestinal (GI) colonization resistance against pathogens, a mechanism through which the commensal intestinal microbiota inhibits colonization by pathogens and opportunistic microbes. The protective mechanism is mediated through various functions, such as nutrient competition, the production of antimicrobial compounds, the modulation of the host immune system, and the maintenance of epithelial barrier function.^5–7^ In addition to these localized functions, the microbiota plays a vital role in the host immune system's induction, training, and function, including the maintenance of regulatory pathways involved in immune tolerance.^8–10^ Notably, when the gut barrier is compromised, the GI microbiome has also been implicated in the development of infections, including those in the lungs, blood, and urine.^11^

Antibiotics can have adverse effects on the commensal microbiota, including a reduction in microbial diversity, loss of beneficial taxa, and selection for antibiotic-resistant genes (ARGs).^12–17^ In addition, antibiotics contribute to the enrichment of MDROs and promote horizontal transfer of resistance genes to other organisms within the microbiota,^18^ leading to the potential emergence of new multidrug-resistant organisms, which may limit the treatment options for bacterial infections. Therefore, studies elucidating protective and deleterious microbiota, resistome characteristics (the collection of ARGs within the microbiota milieu), and antibiotic administration practices leading to the disruption of microbial communities and the emergence of antibiotic resistance are crucial for the development of novel approaches to mitigate AR.^19–21^

Patients with hematological malignancies are at a greater risk of acquiring infections, particularly with AR pathogens.^22^^,^^23^ Infectious complications in patients with hematological malignancies occur frequently in patients with chemotherapy-induced cytopenia. Febrile neutropenia is frequent amongst acute myeloid leukemia (AML) patients, occurring in ~80%−90% of patients, with 20%−40% of those having a microbially documented infection.^24^^,^^25^ In addition to routine fluoroquinolone prophylaxis for all patients receiving induction chemotherapy (IC), the standard of care is to empirically treat patients who develop neutropenic fever with antimicrobials (cefepime, piperacillin‒tazobactam, or carbapenem) based on the increased risk of infection.^26^^,^^27^ It is thought that chemotherapy-induced mucosal damage and intestinal permeability allow the translocation of potentially resistant pathogenic species from the intestinal lumen into the bloodstream, increasing the risk of systemic infection.^28^^,^^29^ The most common bacteria causing infections in AML patients include major CDC AR-threat pathogens such as MRSA, VRE, ESBL-producing or CR-Enterobacteriaceae, such as *E. coli* and *Klebsiella* spp., and MDR *P. aeruginosa.*^25^ Patients receiving cancer therapy, particularly those with AML, are at high risk for GI colonization and subsequent infections with AR pathogens. Monitoring of the GI tract is critical not only for assessing an individual’s risk of infection but also for broader infection control and prevention of transmission in healthcare settings.^30^^,^^31^ Rectal swabs for multidrug-resistant organism (MDRO) surveillance are commonly used to detect colonization by resistant organisms such as VRE, CRE, or ESBL producers.^30^^,^^31^ However, these swabs offer a limited view – often missing critical information such as colonization density, microbial community dynamics, and broader resistome information.^32^^,^^33^ To address these limitations, we used a combination of culture-based techniques and high-throughput sequencing approaches to determine colonization and infectious events caused by CDC-threat AR-pathogens in a cohort of 154 newly diagnosed AML patients receiving IC. By utilizing shotgun metagenomic sequencing (SMS), we were able to comprehensively characterize the baseline (BL) and end-of-study (EOS) GI microbiomes and resistome characteristics to identify the microbial species, functional pathways, and ARG differences associated with AR infection (ARI) and/or AR colonization (ARC) with CDC-threat pathogens.

## Materials and methods

### Ethics statement

This study was approved by the University of Texas MD Anderson Cancer Center (MDACC) Institutional Review Board (PA13-0339 and PA15-0780) and was conducted in full compliance with the guidelines outlined in the Helsinki Declaration of the World Medical Association. All patients enrolled in the study provided written informed consent prior to enrollment.

### Study population and sample collection

Longitudinal stool samples and clinical metadata were collected from two cohorts, comprising a total of 154 newly diagnosed adult AML patients who underwent IC at the MDACC between September 2013 and February 2020 (Table 1). A cohort of patients enrolled from September 2013 to August 2015 (PA13−0339) consisted of 566 stool specimens from 98 AML patients.^34–36^ A BL stool sample was defined as the first stool collected either before the initiation of IC or within one week of the start of IC for each patient. Only one BL sample was collected per patient. Following the BL collection, additional stool samples were obtained every 96 h, as available, and continued until neutrophil recovery, as previously described.^34–36^ An additional cohort of patients was enrolled from January 2015 to February 2020 (PA15-0780), consisting of 216 stool samples from 56 adult AML patients undergoing IC. For this cohort, stool samples were collected at the following intervals: twice a week during 0−4 weeks, once weekly during weeks 5−8 weeks, every two weeks from weeks 8−24 (if available) or until loss to follow-up. The first stool sample collected before receipt of IC or within a week of IC from patients in PA15 was defined as the BL stool sample. EOS stool samples for PA13 were defined as the stool sample collected at the time of neutrophil recovery for each patient (median time of 22 d), whereas for PA15 was defined as the last stool sample collected at the end of the observation period (median time of 26 d). Owing to considerable loss to follow-up for PA15 individuals after the IC inpatient period, the two study EOS sample timepoints were comparable.

**Table 1.** Characteristics of patients used in this study.

Patient characteristics	No. (%)
Patient count	154
Patients with infectious isolates from potential CDC threat species^a^	21 (13.63)
No. of infectious isolates from potential CDC threat species	33
No. of patients with confirmed ARI^b^	8 (5.19)
No. of ARI isolates^c^	12
Patients with colonizing isolates from potential CDC threat species	27 (17.53)
No. of colonizing isolates from potential CDC-threat species	45
No. of patients with confirmed ARC^d^	14 (9.09)
No. of ARC isolates	20
Sex, *N* (%)	
Female	72 (46.75)
Male	74 (48.05)
Unavailable information	8 (5.19)
Chemotherapy intensity, *N* (%)	
High	82 (53.24)
Low	64 (41.55)
Unavailable information	8 (5.19)
Antibiotic administration, *N* (%)^e^	
Piperacillin-Tazobactam	40 (25.97)
Cephalosporin	103 (66.88)
Carbapenem	72 (46.75)
Unavailable information	4 (2.59)

^a^ Potential CDC threat species include methicillin-resistant *Staphylococcus aureus* a(MRSA), multi-drug-resistant *Pseudomonas aeruginosa* vancomycin-resistant *Enterococcus* (VRE), extended-spectrum beta-lactamase producing Enterobacteriaceae (ESBL), and carbapenem-resistant Enterobacteriaceae (CRE).

^b^ Confirmed AR-infection with CDC AR-threat pathogens including VRE, MRSA, CRE, ESBL-producing Enterobacteriaceae, and MDR *Pseudomonas aeruginosa.*

^c^ Often the same patient would have multiple ARI or ARC isolates.

^d^ Confirmed colonization with an AR-pathogen deemed to be a CDC-AR threat pathogen.

^e^ Patients received the broad-spectrum antibiotics noted for at least 48 h during the study period.

### 16S rRNA sequencing and analyses of the stool samples

Genomic DNA was extracted from stool samples by utilizing the QIAamp Fast DNA Stool Mini Kit (Qiagen), with some modifications to the established kit protocol by introducing an additional bead-beating lysis step. Each stool sample was added to a tube containing a 3.2-mm steel bead, approximately 150 mg of zirconium beads, and the lysis buffer. The stool samples were then homogenized with a bead-beater for 8  min, at a speed of 3800 rpm (BioSpec) for DNA isolation. The 16S V4 amplicon libraries were generated, and Illumina MiSeq sequencing of the 16S rRNA gene V4 region was performed using a 2 × 250 bp paired-end protocol.^34–36^ The reads were merged, dereplicated, and length-filtered utilizing VSEARCH. Following denoising and chimera calling using the UNOISE3 commands,^37^ the unique sequences, or zero-radius zOTUs, were taxonomically classified using Mothur^38^ with the SILVA database version 138. Alpha and beta diversity metrics were generated in QIIME 2. The 16S rRNA sequences of the PA13 stool samples were previously published and deposited in the NCBI Sequence Read Archive under the Bioproject IDs PRJNA352060 and PRJNA526551.^34–36^ The 16S rRNA sequences for the PA15 cohort are also deposited in the NCBI Sequence Read Archive under the Bioproject number PRJNA1124986.

### **Determination of AR-threat colonization and infection**

The 16S rRNA sequencing of all longitudinal stool samples were analyzed to determine which stool samples contained >3% of their 16S rRNA reads mapping to the genera representing potential AR-threat pathogens such as VRE, CRE or ESBL, MDRP, and MRSA. Samples with >3% of their reads mapping to Enterobacteriaceae, *Escherichia*, *Enterobacter*, *Klebsiella*, or *Pseudomonas* were streaked on HardyCHROM™ CRE or HardyCHROM™ ESBL (Hardy Diagnostics). Stools with >3% of their reads mapping to *Enterococcus* were streaked on HardyCHROM™ VRE media (Hardy Diagnostics), while those with *Staphylococcus* reads were streaked on HardyCHROM™ MRSA (Hardy Diagnostics). Colonies that grew on selective media were then streaked on BBL Trypticase Soy Agar with 5% sheep blood (BD Bioscience) to isolate single/individual colonies, which were subsequently stored at −80 °C. Matrix-assisted laser desorption/ionization time-of-flight mass spectrometry (MALDI-TOF) was performed on purified colonies for identification of a specific bacterium at the species level (Bruker MALDI Biotyper). Following bacterial identification, VITEK2 (Biomerieux) was used to determine the antibiotic susceptibilities of bacterial isolates utilizing AST-GN69 and AST-XN06 cards for Gram-negative bacteria and AST-GP75 cards for Gram-positive bacteria. If an isolate from an individual stool sample was confirmed to be CRE, ESBL-producing Enterobacteriaceae, VRE, MRDP, or MRSA, the patient was considered to have confirmed ARC.

Furthermore, any infectious bacterial isolates collected by the clinical microbiology lab at the MDACC while the enrolled AML patients were in the study were stored at −80 °C. The clinical microbiology lab at MDACC determined the bacterial identification and antimicrobial susceptibilities of these isolates. Infections were classified by two-independent investigators and defined as microbiologically documented infection (MDI) utilizing consensus definitions for neutropenic hosts and were considered only when there are signs and symptoms of infection with microbiologic culture confirmation in the setting of febrile neutropenia.^27^^,^^39^ If the clinical microbiology lab confirmed the microbially defined infection isolate to be CRE, ESBL-producing Enterobacteriaceae, VRE, MRDP, or MRSA, the patient was considered to have a confirmed ARI.

### **Whole genome sequencing and analysis of bacterial isolates causing AR-threat colonization and infection**

DNA was extracted from individual bacterial isolates via the MasterPure Gram-positive DNA purification kit (Lucigen). Whole-genome sequencing (WGS) was performed using the NovaSeq S4 platform with a 150-bp paired-end read protocol. The genomes of the bacterial isolates are deposited in the NCBI Sequence Read Archive under the Bioproject PRJNA1129516. The bacterial isolate reads were downsampled to 6 million read pairs per sample using Seqtk (v1.3) and assembled in SPAdes (v3.14.1) under the "--isolate" setting. The isolate assemblies were annotated on the Bacterial and Viral Bioinformatics Resource Center (BV-BRC) using the RAST tool kit (RASTtk) pipeline.^40^ A comprehensive genome analysis was performed using BV-BRC to access genomic information from individual bacterial isolates and examine the virulence factors and antimicrobial resistance genes.^40^ The comprehensive antibiotic resistance database (CARD) was used to identify ARGs, while the virulence factor database (VFDB) was used to examine virulence factors. Multilocus sequence typing (MLST) was performed using the PubMLST website to characterize the bacterial isolates, except for *K. pneumoniae*, for which the Pasteur MLST database and software were used. The Achtman MLST scheme was applied to *E. coli*.

### **Stool DNA extraction, shotgun metagenomics, and bioinformatic analysis**

Genomic DNA was obtained from stool samples with a DNeasy Blood & Tissue kit (Qiagen, CA, USA) according to the manufacturer’s instructions, with an additional bead-beating step for lysis and the addition of InhibitEX buffer (Qiagen). Briefly, 150 mg of frozen feces from each sample was added to 500 µL of sterile InhibitEx Buffer (Qiagen, Valencia, CA, USA), and 150 mg of silicon beads (Lysing Matrix B, MP Biomedical) and a suitable amount of hard tissue grinding mix 2.4 mm metal beads (VWR, Radnor, PA, USA), were added into a sterile tube. The tubes were shaken at 4.5 m/s for 4 min (MP Biomedical FastPrep-24 Classic). The resulting suspension was vortexed, heated at 95 °C for 7 min, and centrifuged at 15,000 rpm for 3 min. Patients with missing stool samples at either the BL or EOS were excluded from the metagenomic analyses. Furthermore, samples that did not meet the standards for DNA quality or metagenomic sequence quality were also excluded. SMS was performed on 181 stool samples from 123 AML patients using the NovaSeqS4 platform with 150-bp paired-end sequences. The SMS sequences for the PA13 and PA15 stool samples were deposited in the NCBI Sequence Read Archive under the Bio project PRJNA1129514 and PRJNA1128111, respectively. Megahit (v1.2.8) and metaSPAdes (v3.14.1) were used to assemble the shotgun metagenomic sample reads. The raw reads were subjected to quality control with fastp. Megahit shotgun metagenomic assemblies were binned and annotated on BV-BRC using the RAST Binning Service (RBS). MetaPhlAn2 (v2.8.1) was used to determine the relative abundance of bacterial taxa, and QIIME2 was used to calculate the *α*-diversity metrics.

Assemblies were then taxonomically binned by BugSeq (v4.0), as previously described.^41^ In brief, contigs were aligned against a curated reference sequence database using minimap2, and alignments were parsed for query coverage and absolute nucleotide identity (ANI) against reference sequences; these factors were combined to yield taxonomic classifications for each contig based on established ANI and coverage thresholds. Taxonomic bins were searched for genomic predictors of antimicrobial resistance (AMR) using BugSeq’s AMR analysis (v4.0). In brief, contigs were searched for proteins from a curated database containing over 6500 protein sequences associated with AMR. A protein search was performed with threshold alignment and a hidden Markov model to identify both gene allele and family. Taxon-specific models for phenotypic AMR prediction, which include single-nucleotide variants and other predictors of resistance (e.g., indels), were applied for over 50 bacterial species. Taxonomic binning data and AMR predictions were combined into reports for downstream analysis.

SMS were then run through the HUMAnN 2.0 (v2.8.2) pipeline for functional profiling using the Uniref90 database and default settings.^42^ Gene and pathway abundances were normalized for sample sequencing depth in copies per million and were regrouped into KEGG Orthology terms. Heatmaps were made using the *pheatmap* package in R. Virulence and antibiotic resistance genes (determined by BV-BRC) were compiled into individual spreadsheets according to their presence (“1”) and absence (“0”). The samples were annotated for their species and the colonization or infection status using the annotation function in the *pheatmap* package.

### **Statistical and network analysis**

Differences in the alpha diversity between the AR outcome groups were assessed using the Mann‒Whitney U test in GraphPad Prism version 10.0.0. Benjamini‒Hochberg (BH) corrections were performed to decrease the false discovery rate. Beta diversity differences between AR outcome groups were assessed using Bray–Curtis dissimilarity and visualized using principal coordinate analysis (PCoA) plots in the vegan and cluster packages in R. Non-parametric permutational multivariate analysis of variance (PERMANOVA) was used to test sample clustering in beta diversity analysis with 999 replicate permutations using the Adonis function in the vegan package in R.

For the statistical analysis of differences in the relative abundance of specific taxa between outcome groups, the Mann‒Whitney U test was performed on centred log-transformed (CLR-transformed) data. Before statistical analysis, relative abundance data were filtered to include only taxa with an average 1% relative abundance and 25% prevalence across the total number of samples. A Welch test was performed to identify functional pathways at baseline that were significantly abundant in each patient group (ARI, ARC, and ARI and/or ARC). A clustered heatmap of the KEGG pathways within each group was created in R using the *heatmap.2* function, and the top 10% of pathways were organized by effect size. Cohen's D was used to determine the effect size.

To compare the number of resistance elements between the BL and EOS groups, we first assessed the normality of the data using QQ-plots and density plots, which were generated with the ggpubr package in R, in addition to performing the Shapiro‒Wilk test. When the data were found to be normally distributed, a student’s t-test was performed to compare the number of resistance elements between the BL and EOS groups. The Mann‒Whitney U test was used for non-parametric outcome group comparison, including ARI vs. No-ARI, ARC vs. No-ARC, and any-AR event vs. No-AR event. The odds ratio was utilized to compare the odds of developing ARI, ARC, and any AR event based on the presence of antimicrobial-resistant gene classes in BL stools. All tests were conducted using the R (version 4.3.0). Statistical significance was defined as *p*-values less than 0.05.

A correlation-based network was constructed to explore the relationships between bacterial species and ARGs. Two datasets were used, one containing bacterial species relative abundance data and the other with ARG count data. A pairwise Spearman correlation matrix was generated in R to assess the relationships between variables within these two datasets. To optimize the network, variables with correlations with absolute values greater than 0.3 in addition to statistically significant (*p *< 0.01) were imported into Gephi for network visualization and analysis. The schematic representation of the experimental design was generated using BioRender.

## Results

### **Patient characteristics**

**Figure 1.**
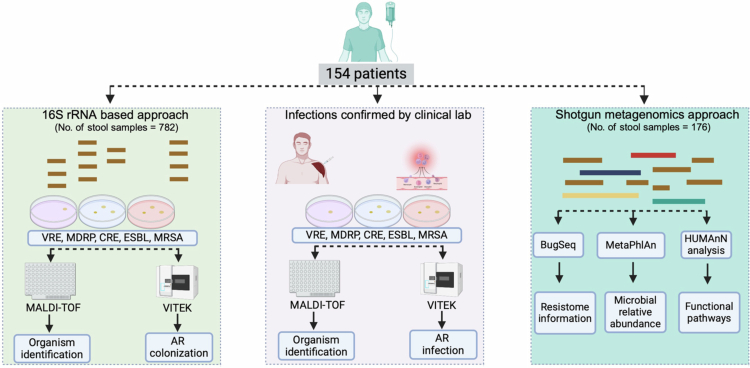
Patient sampling and determination of AR-events. A schematic representation of the workflow of adult AML patients enrolled in the study and information regarding their AR-threat determination and genomic sequencing analysis.

A total of 154 AML patients provided 782 stool samples starting from BL until the EOS (median 23 d for all samples combined). The characteristics of these patients are provided in Table 1. The majority of AML patients in our cohort received empirical antibiotics for the treatment of febrile neutropenia according to the IDSA guidelines for antimicrobial use in neutropenic patients with cancer during IC,^27^ with ~67% of patients receiving cephalosporins, ~47% of patients receiving carbapenems, and ~26% of patients receiving piperacillin‒tazobactam for at least 48 h (Table 1). 16S rRNA Illumina MiSeq sequencing of 782 stool samples provided relative quantification of the bacterial genera present in each sample (Figure 1). A total of 88 stool samples from 84 AML patients were found to have >3% abundance of the CDC-threat genera *Staphylococcus* (18), *Enterococcus* (31), *Pseudomonas* (6), or *Enterobacteriaceae* (33) by 16S rRNA V4 sequencing. The stool samples were tested on selective and differential VRE, ESBL, CRE, and MRSA media. A total of 45 colonizing bacterial isolates from potential CDC threat species were isolated from 27 patients; however, only 20 of the colonizing isolates were confirmed to meet our definition of AR-threat colonization from 14 patients (Table 1 and Supplemental Table 1).

Similarly, 33 infection isolates were collected from 21 patients caused by species known to be CDC AR threats. Twelve of the isolates met our definition of AR threat infection, representing 8 patients (Table 1 and Supplemental Table 2). In addition to 16S sequencing data, we obtained SMS data from BL and EOS stool samples (*n* = 176) from 123 AML patients (Figure 1). Among them, 105 patients with BL stool samples, and 71 patients with EOS stool samples; these samples passed the DNA and sequencing quality standards (Table 2). As expected, the frequency of ARI was greater among female patients (Table 2), reflecting their increased susceptibility to adverse events and drug-associated toxicity with treatment for hematologic malignancies.^43^^,^^44^

**Table 2.** Characteristics of patients used for shotgun metagenomics sequencing.

		ARI^b^^,^^c^				ARC^d^				ARI and/or ARC			
Patient characteristics	No. of patients	Yes	No	*p*-Value	Odds ratio (CI)	Yes	No	*p*-Value	Odds ratio (CI)	Yes	No	*p*-Value	Odds ratio (CI)
Patient count	123^a^	8	115			14	109			19	104		
Baseline	105	8	97			11	94			16	89		
EOS	71	4	67			10	61		12	59			
Sex, *N* (%)				0.031^f^	0.125			0.744	1.438			0.787	0.826
Female	57 (46.34)	7	50			4	53			8	49		
Male	59 (47.20)	1	58			6	53			7	52		
Unavailable	7 (5.69)	0	7			4	3			4	3		
Chemotherapy intensity				1.000	0.764			0.510	0.534			1.000	0.846
High	66 (52.84)	5	61			7	59			9	57		
Low	51 (41.46)	3	48			3	48			6	45		
Unavailable	6 (4.87)	0	6			4	2			4	2		
Antibiotic administration *N* (%)^e^													
Cephalosporin	82 (66.67)	6	76	0.718	1.534 (0.259−16.240)	6	76	0.509	0.603 (0.142−2.676)	10	66	0.778	0.812 (0.244−2.947)
Carbapenem	50 (40.65)	3	47	1.000	0.869 (0.129−4.716)	5	45	0.755	1.257 (0.285−5.293)	7	43	0.791	1.156 (0.338−3.794)
Piperacillin-Tazobactam	27 (21.95)	4	23	0.069	3.942 (0.680−22.890)	4	23	0.253	2.216 (0.436−9.681)	6	21	0.116	2.436 (0.651−8.434)

^a^ Listed are 123 patients who had shotgun metagenomics data available.

^b^ Confirmed AR-infection with CDC AR-threat pathogens including VRE, MRSA, CRE, ESBL-producing Enterobacteriaceae, and MDR *Pseudomonas aerugiosa.*

^c^ Often the same patient would have multiple ARI or ARC isolates.

^d^ Confirmed colonization with an AR- pathogen deemed to be a CDC-AR threat pathogen.

^e^ Patients received the broad-spectrum antibiotics noted for at least 48 h during the study period, and was only included if prior to event, or censored at 8 weeks in individuals with no event.

^f^ Fisher’s exact test was performed to determine the *p*-value. A *p*-value < 0.05 was considered statistically significant.

### Molecular epidemiology of AR-threat colonization and infection isolates

The AR colonizing and infecting *E. coli* strains consisted of three ST44, three ST167, one ST361, and one ST131, all of which are sequence types frequently collected in the clinical setting and associated with MDR (Supplemental Table 3).^45–48^ Similarly, two *P. aeruginosa* isolates were represented by ST111, two by ST179, and the remaining two by ST298, all of which are high-risk epidemic MDR clones of *P. aeruginosa.*^49^ MRSA isolates included the hypervirulent epidemic clones ST5 and ST105,^50^ and all three colonizing *K. pneumoniae* strains were represented by the MDR hypervirulent clone ST29.^51^^,^^52^ Among the VRE isolates, multiple STs were identified (17, 18, 665, and 1516), with two isolates belonging to hospital-adapted CC17 strains.^53^

**Figure 2.**
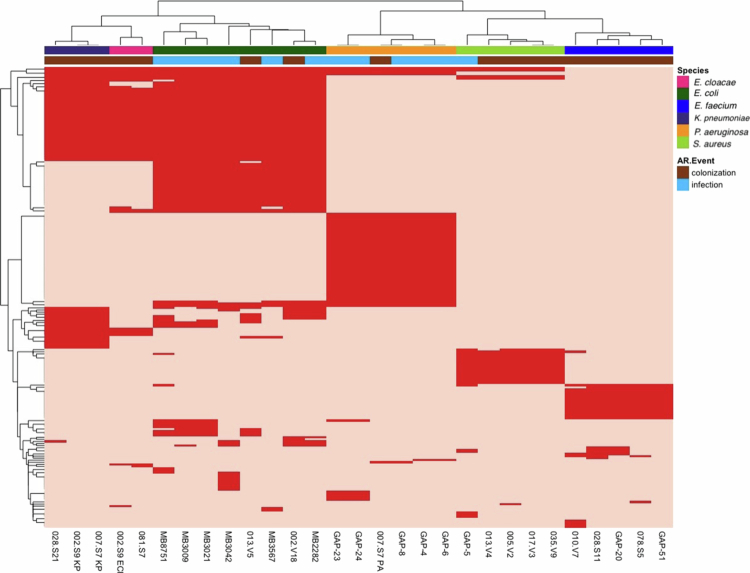
Presence of antibiotic resistance genes in AR-threat infectious and colonizing isolates. Dark red in the heatmap indicates the presence of the gene, and light red indicates its absence. AR-colonizing and infectious isolates are denoted by the secondary annotation bar at the top of the heatmap, with colonizing isolates in brown and infectious isolates in blue. The bacterial species can be identified by the primary annotation bar at the top of the heatmap. The heatmap utilizes Euclidean hierarchical clustering.

ARGs carried by the ARC and ARI bacterial isolates were analyzed. When clustered by the ARG profile, the ARC and ARI isolates were segregated perfectly by species (Figure 2). Notably contributing to the carbapenem-resistant and extended-spectrum *β*-lactamase-producing phenotypes, all *K. pneumoniae* strains contained the presence of TEM-1 *β*-lactamase, CTX-M-15 *β*-lactamase, the SHV-187 *β*-lactamase, OXA-1 *β*-lactamase, the OmpK35 porin, and the AcrAB-TolC efflux pump. The CTX-M−55 *β*-lactamase was detected in 4 *E. coli* isolates, TEM-1 *β*-lactamase in 2 *E. coli*, OXA-1 *β*-lactamase in 4 *E. coli* isolates, whereas CTX-M-15 was detected in 3 *E. coli* isolates. All *P. aeruginosa* isolates contained OXA-50, as well as multiple multidrug efflux systems, including MexAB-OprM and MexEF-OprN. Two of the *P. aeruginosa* isolates also carried CARB-3 and OXA-9. In addition to the *van* operon, *E. faecium* revealed common ARGs genes associated with resistance to macrolides (*ermB*) and the aminoglycosides AAC(6')-Ie-APH(2'')-Ia, AAC(6')-Ii, and APH(3')-IIIa. All the MRSA strains contained the *mec* operon, while only one contained *blaZ.* All other ARGs detected amongst ARC and ARI isolates are listed in Supplemental Table 4.

**Figure 3.**
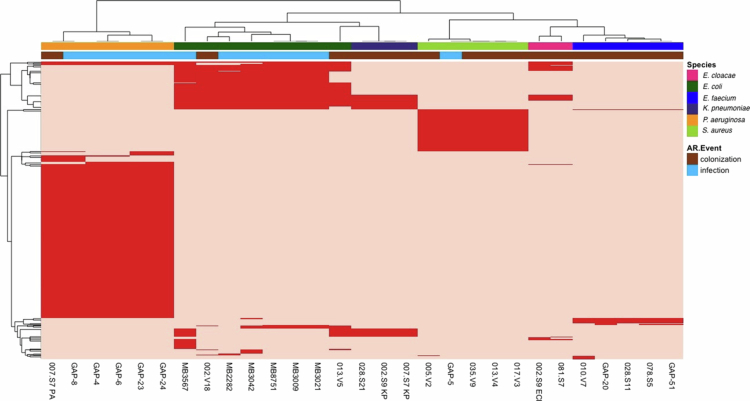
Presence of virulence genes in AR-threat infectious and colonizing isolates. The dark red in the heatmap indicates the presence of the gene, and the light red indicates its absence. AR-colonizing and infectious isolates are denoted by the secondary annotation bar at the top of the heatmap, with colonizing isolates in brown and infectious isolates in blue. The bacterial species can be identified by the primary annotation bar at the top of the heatmap. The heatmap utilizes Euclidean hierarchical clustering.

Similar to the antibiotic resistance genes, Euclidean hierarchical clustering of virulence genes grouped the isolates perfectly by species, and the majority of strains were grouped by colonizing and infectious isolates within species (Figure 3, Supplemental Table 5). All *P. aeruginosa* strains contained the five distinct operons comprising the T3SS regulon, including the *pscN* to *pscU*, the *exsD-pscB* to *pscL* operons, the *popN-pcr1-pcr2-pcr3-pcr4-pcrD-pcrR* operon, the *pcrGVH-popBD* operon and finally the *exsCEBA* operon. The five operons are important in the biogenesis and translocation of type III secretion.^54^ All *P. aeruginosa* strains also contained the T4SS low-abundance minor pilins PilVWXE, but only two had the major pilin subunits, PilA. They also have H1-T6SS system machinery and *tse* effector genes.

All *E. coli* isolates contain operons involved in biofilm formation, including the *csgBAC* operon (which encodes curli structural subunits) and the *csgDEFG* operon (which encodes the CsgD transcription regulator and the CsgEFG curli-specific transport system).^55–59^ All the isolates also contained the siderophore enterobactin (*ent*) operon, which is important for iron acquisition type I fimbriae (*fim* genes), the “*E. coli* common pilus” (ECP), which is composed of a major pilin subunit encoded by the *ecpA*/yagZ, and its associated operon (*ecp/yag* genes), and the machinery for T2SS and pseudopilin biogenesis (*gsp* genes) (Supplemental Table 5).^60–63^ Similarly, *K. pneumoniae* contained type I fimbriae, the ECP operon, and enterobactin but also contained the operon encoding the siderophore yersiniabactin (*ybt* genes).^64-67^

All the ARI and ARC bacterial isolates of *E. faecium* carried the collagen adhesins *acm* and *scm*, as well as enterococcal surface protein (*esp*), all of which are important for host tissue adherence.^68–70^ Three of the five *E. faecium* isolates contained the gene encoding another collagen-binding factor, *ecbA.*^71^ All five *S. aureus* also contained the same virulence factors, regardless of colonization or infection status. This included the *cap8* operon involved in capsular polysaccharide production, the gene encoding the fibrinogen binding protein *clfA*, genes involved in the ESS secretion pathway important for host invasion (*esx* and *esa* genes), the *ica* operon involved in exopolysaccharide biosynthesis important for biofilm formation, the Panton-Valentine leucocidin, and the surface adhesin operon *sdrCDE.*^72–77^

### Gut microbiome and resistome features at baseline and end of study

**Figure 4.**
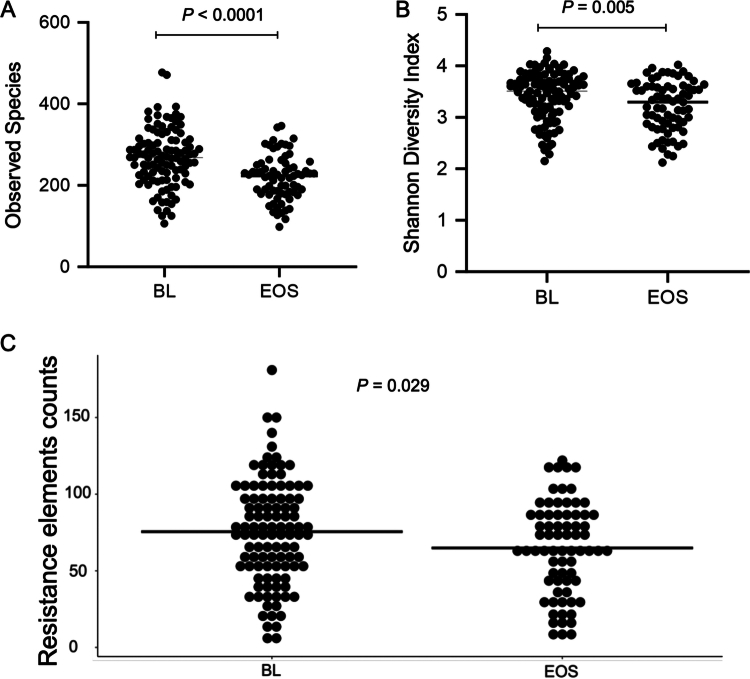
Comparisons of *α*-diversity metrics and the number of resistance elements between baseline and the end of the study among AML patients receiving induction chemotherapy. *α*-Diversity in AML patients was measured by (A) observed OTUs and (B) the Shannon diversity index, while the number of unique resistance elements (C) was measured for baseline (BL) and end-of-study (EOS) stool samples. *p*-Values generated by Mann‒Whitney <0.05 were considered statistically significant.

Using SMS data, we analyzed the BL and EOS stool samples from 123 AML patients to assess whether the course of treatment, including chemotherapy and antibiotics, was associated with changes in microbial diversity and microbial composition at the species level in AML patients during IC. Consistent with our previous studies, which utilized 16S data, we observed a significant reduction in the observed species (*p* <  0.0001) and Shannon diversity index (*p =* 0.005) at the EOS compared to BL stool samples (Figure 4). Additionally, several bacterial taxa presented statistically significant differences in abundance between the BL and EOS, with 50% of the taxa at the genus level and 36% at the species level identified as differentially abundant (Supplemental Table 6). Among them, the genera *Bacteroides*, *Bifidobacterium*, *Blautia*, *Coprococcus*, *Ruminococcus*, and *Roseburia* were significantly greater at BL compared to EOS. This included their respective species *Bacteroides massiliensis, Bacteroides ovatus*, *Bacteroides stercoris*, *Bacteroides thetaiotaomicron*, *Bacteroides vulgatus*, *Blautia producta*, and *Ruminococcus gnavus*. Similarly, *Akkermansia muciniphila* was also more abundant in BL stools.

Next, we explored the changes in the number and types of resistance elements between the BL and EOS stool samples. BL stool samples had significantly greater (*p =* 0.029) resistance elements (mean = 75.51, SD = 33.77) compared to the EOS stool samples (mean = 64.88, SD = 29.83) as illustrated in Figure 4C. Additionally, there was a greater number of predicted antibiotic resistance genes (ARGs) encoding glycopeptides, lincosamide, lincosamide/macrolide, tetracycline, and lincosamide/oxazolidinones/streptogramin at baseline (Supplemental Table 7).

### Association of baseline microbiome characteristics with ARI

**Figure 5.**
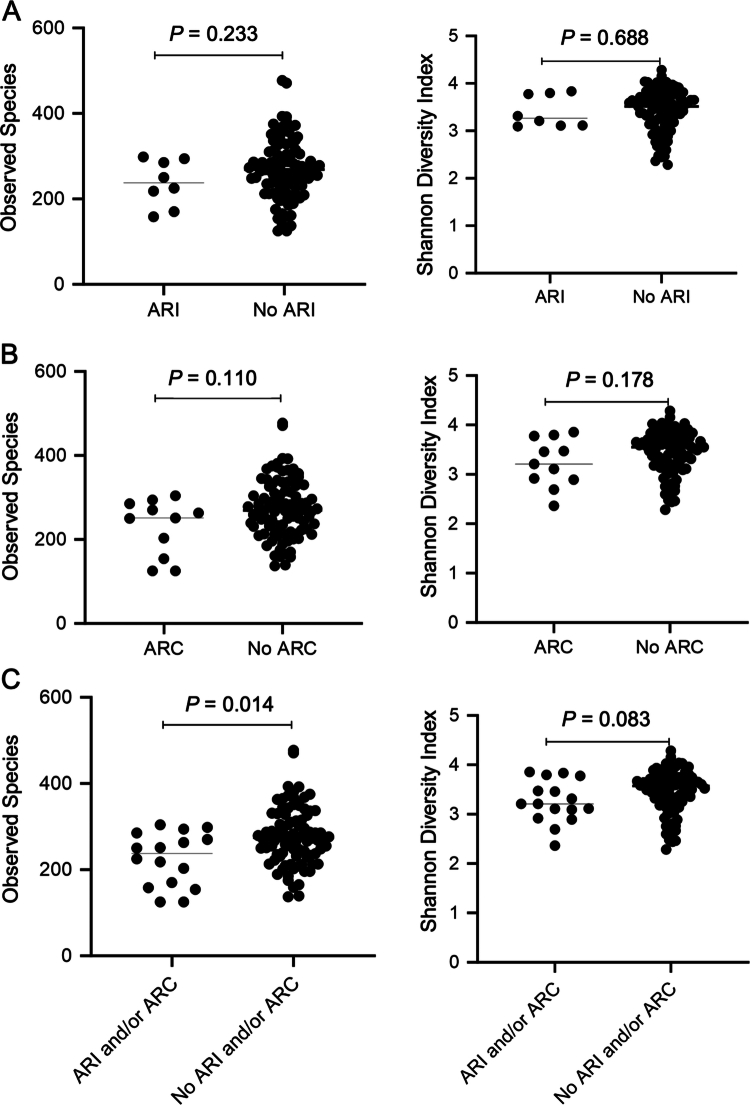
Comparisons of *α* diversity metrics among AML patients who did and did not develop AR threat events. The observed species (on the left) and the Shannon diversity index (on the right) are shown for adult AML patients at baseline, comparing those who developed (A) ARI, (B) ARC, and (C) either ARI or ARC to those who did not.

We next investigated whether the BL gut microbiome is associated with the ARI and/or ARC. Through SMS data, the association between BL gut microbiome characteristics and the subsequent development of infection with AR-threat pathogens in AML patients were assessed. Among the 105 AML patients with a BL sample passing SMS quality standards, 8 patients developed an infection with an AR-threat pathogen (Table 2). We first compared *α* diversity measures amongst patients who did and did not experience ARI. There was no statistically significant difference in the observed species or Shannon diversity of BL stool between patients who developed or did not develop ARI (Figure 5A).

We next investigated differentially abundant taxa in subjects who experienced ARI with a CDC-threat pathogen. Although unadjusted analysis showed a greater abundance of *Clostridium* (*p =* 0.038) and *Clostridium boltae* (*p =* 0.033) in the BL stool samples of patients who experienced ARI, these differences did not remain significant after adjusting for multiple comparisons (adj. *p =* 0.545 and 0.659, respectively; Supplemental Table 8). Similarly, we did not find any ARGs that were significantly associated with increased or decreased odds of ARI events (Supplemental Table 10).

**Figure 6.**
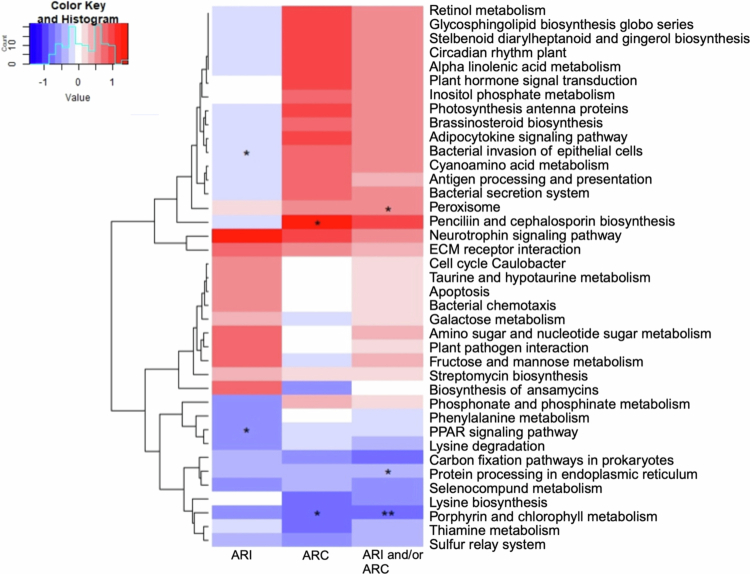
Baseline functional pathway differences amongst AML patients developing AR-threat events during induction chemotherapy. The top 10% differential functional pathways based on the effect size at baseline amongst patients developing AR threat events are visualized. Blue indicates a negative effect size (Cohen’s D), while red represents a positive effect size amongst individuals developing the denoted AR-threat event. White indicates no effect between the two groups. An asterisk (*) indicates significant differences in pathways with a *p* <  0.05., while two asterisks (**) denote *p* < 0.01.

Additionally, the changes in pathway activities between patients who experienced ARI versus those who did not experience any ARI were also compared (Figure 6). Twenty-six of the top 40 pathways had a negative effect size, and 2 of them, including PPAR signaling pathways (*p =* 0.019, Cohen’s D = −0.485) and bacterial invasion of epithelial cell pathways (*p =* 0.048, Cohen’s D = −0.226), were significantly different between patients who did and did not experience ARI. Furthermore, 10 of the top 40 pathways had a moderate to large positive effect size > 0.5. Among them, the neurotrophin signaling pathway (Cohen’s D = 1.353), the ECM receptor interaction (Cohen’s D = 0.857), and the biosynthesis of ansamycins had a large effect size (Cohen’s D = 0.864) (Figure 6); however, these effects were not statistically significant.

### Association of baseline microbiome characteristics with ARC

Next, we assessed the relationship between the BL gut microbiome and the colonization with AR-threat pathogens. Among those 105 patients with a BL sample, 11 patients were colonized with a CDC AR-threat pathogen (Table 2 and Supplemental Table 1). There was no statistically significant variation observed in BL stool *α*-diversity or observed species between patients who were and were not colonized with AR-threat pathogens (Figure 5B). The comparisons between patients who were and were not colonized with AR-threat pathogens demonstrated variations in the relative abundance of several genera and species at BL (Supplemental Table 8). For example, *Methanobrevibacter* (*p =* 0.009) and *Roseburia* (*p =* 0.008) were more abundant in colonized patients. However, none of these differences remained statistically significant when the *p*-values were adjusted for false discovery (adj. *p* = 0.155).

A comparison of the mean abundance of ARGs at BL showed genes encoding resistance to beta-lactam resistance (*p* = 0.045; adj. *p* = 0.420) and tetracycline resistance (*p* = 0.036; adj. *p* = 0.4200) were greater in patients who developed ARC, although the difference was not statistically significant when the *p*-values were adjusted (Supplemental Table 9). Notably, the presence of genes predicted to encode aminoglycoside resistance (OR = 0.09, *p* = 0.04) and trimethoprim resistance (OR = 0.124, *p* = 0.01) at BL was associated with decreased odds of colonization with an AR-threat pathogen during IC (Supplemental Tables 3 and 10).

When comparing pathways amongst patients who were or were not colonized with AR threats, four pathways showed a moderate or greater negative effect (Cohen’s D <  −0.5) out of the top 40 pathways. Among them, porphyrin and chlorophyll metabolism were statistically different in patients with ARC (*p =* 0.012, Cohen’s D = −0.852; Figure 6). Eighteen pathways exhibited a moderate or large positive effect (Cohen’s D > 0.5, including the penicillin and cephalosporin biosynthesis (*p =* 0.028, Cohen’s D = 1.442), which was statistically enriched in ARC patients.

### Association of baseline microbiome characteristics with any AR-threat event

Next, we combined the subjects with ARI and/or ARC to determine whether the BL stool microbiome could be associated with any AR event. A significant difference in the observed species was detected, where the bacterial richness was significantly lower in patients with any AR event than in those with no AR event at BL (*p* = 0.014); however, no significant differences in the Shannon diversity index were observed (*p =* 0.083; Figure 5C). Several bacterial taxa presented lower relative abundances at BL in subjects who experienced any AR risk event than in those who did not (Supplemental Table 8). This included the genera (and species within those genera) *Faecalibacterium* (*p =* 0.043; adj. *p =* 0.175), *Collinsella* (*p* = 0.028; adj. *p =* 0.175), *Methanobrevibacter* (*p =* 0.003; adj. *p =* 0.089), *Dorea* (*p =* 0.036; adj. *p =* 0.175), *Roseburia* (*p =* 0.013; adj. *p =* 0.175), and *Eggerthella* (*p* = 0.028; adj. *p =* 0.175). The differences in relative abundance became nonsignificant when corrected for false discovery.

Among the pathways that had differential effects in patients with any AR event, three pathways had a moderate to large negative effect (Cohen’s D <−0.5, Figure 6). This included porphyrin and chlorophyll metabolism (*p =* 0.006, Cohen’s D = −0.757) and protein processing in the endoplasmic reticulum (*p =* 0.022, Cohen’s D = −0.472), which had a significant and nearly moderate effect. In contrast, 14 pathways indicated a moderate to large positive effect (Cohen’s D > 0.5), where the peroxisome pathway was significantly enriched in patients with any AR threat (*p* = 0.041, Cohen’s D = 0.579).

### Changes in the gut microbiome features over time amongst patients with AR-threat events

We next sought to determine whether individuals who experienced AR-threat events experienced more microbiome disruption over time than those who did not experience any AR-threat events. Among patients without AR risk events, we observed a significant decline in both observed species richness (*p* < 0.0001) and Shannon diversity (*p =* 0.009) from BL to EOS suggesting a microbiome shift during treatment (Supplemental Figure 1). In contrast, no significant differences in *α* diversity were observed in patients who experienced an AR risk event. Furthermore, Bray‒Curtis distances were used to identify any potential differences between BL and EOS in the gut microbiome among patients who experienced AR events. The principal coordinate analysis demonstrated that there were no significant differences in *β* diversity between BL and EOS in the patients who did and did not experience any AR threats (*p =* 0.859; Supplemental Figure 2).

Upon categorizing patients on the basis of AR threat events, we observed several statistically significant changes in bacterial taxa between BL and EOS among patients who did not experience an AR threat event. Specifically, significant reductions were detected in *Bacteroides*, *Barnisiella*, *Bifodobacterium*, and *Ruminococcus* (all adj. *p < *0.001; Supplemental Table 11). In contrast, a significant loss of *Lactobacillus* (adj. *p =* 0.026) was unique to patients who experienced an AR event.

### Network analysis of baseline microbiome and antibiotic features

**Figure 7.**
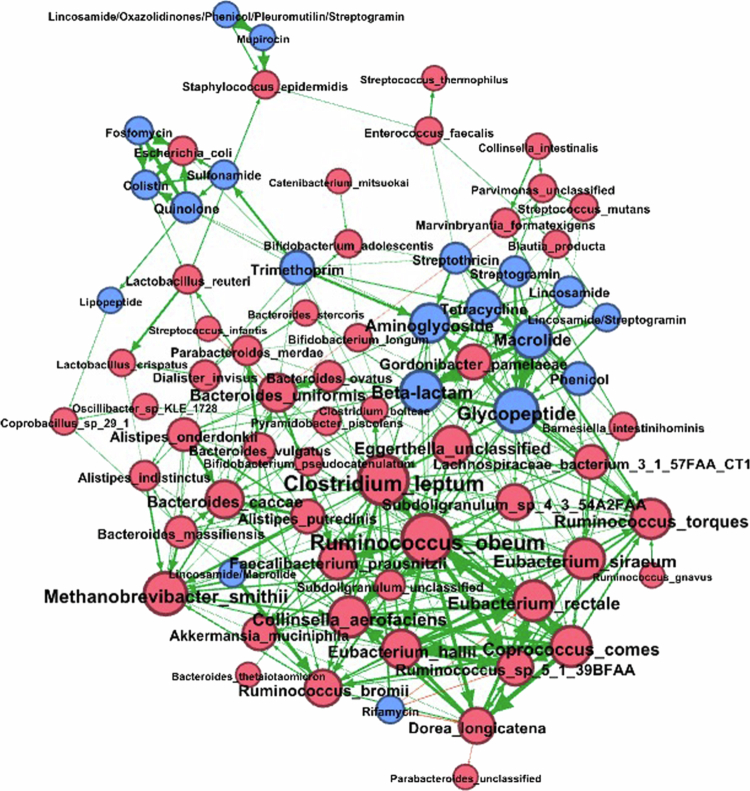
Network analysis of the gut microbiome and resistome features at baseline. A pairwise Spearman rank correlation matrix was generated in R and visualized in Gephi to assess the relationships between bacterial species and ARGs at baseline. Statistically significant bacterial species are represented by red nodes, and ARGs are represented by blue nodes. Positive correlations (*r* > 0.03) are represented by green arrows, while negative correlations (*r* < −0.03) are represented by red arrows.

Finally, a correlation-based network was constructed to explore the relationships between bacterial species and ARGs (Figure 7). The network analysis showed a modularity value of 0.420, indicating the presence of seven distinct communities or subgroups within the network. The calculation of betweenness centrality revealed the highest value of 0.03, which was observed for *Clostridium leptum*. This finding demonstrated that no single entity or node is central to the network; instead, connectivity and influence are relatively evenly distributed among multiple nodes. Other bacterial nodes in the network, such as *Ruminococcus obeum and Bacteroides uniformis* species, along with connected ARGs, such as *β*-lactams, aminoglycosides, and glycopeptides, appeared as other important modules. The vast majority of the correlations (98%) were positive (green arrows) between species and ARGs.

## Discussion

While our previous studies have investigated the association of the baseline gut microbiome with infectious outcomes during chemotherapy, there remains a notable paucity of data regarding the association of gut microbiome features with infection and/or colonization with AR pathogens in AML patients.^35^ Using SMS, we have improved upon previous 16S rRNA-based studies by providing a comprehensive assessment of all species, genes, and pathways present.^34–36^

Previous studies from our lab and others reported a significant decline in *α* diversity over time in patients treated with chemotherapy and/or antibiotics.^34^^,^^35^^,^^78^^,^^79^ In this study, we observed a similar reduction in microbial diversity over time following intensive chemotherapy and/or antibiotic treatment. Notably, the depletion of beneficial genera such as *Roseburia*, *Blautia*, *Barnesiella*, *Bifidobacterium*, and *Akkermansia* may have contributed to this loss of diversity (Supplemental Table 6). Particularly concerning is the reduction in bacteria, such as butyrate-producing *Roseburia* and *Blautia*, which play critical roles in maintaining intestinal barrier function. Dunn et al. examined the impact of antibiotic and antifungal treatment on the microbiome composition of pediatric leukemia and lymphoma patients undergoing chemotherapy and also showed a reduction in the relative abundance of *Blautia*, as well as *Anaerostipes*, *Dorea*, and *Faecalibacterium.*^78^ Similarly, a previously published study involving pediatric AML patients revealed a significant decrease in the number of anaerobic bacteria, including *Faecalibacterium prausnitzii* and *Bacteroides vulgatus*, and an increase in the abundance of enterococci in the gut following treatment with prophylactic antibiotics and chemotherapy.^80^ Although we did not establish any significant increases in specific species between BL and EOS we did observe a consistent decrease in *Bacteroides vulgatus* across all patients.

The shift in microbial composition following chemotherapy and antibiotic treatment is particularly relevant given the observed changes in the gut resistome, with BL samples showing a greater number of unique resistance elements compared to those at the EOS (Figure 4). At BL, higher microbial diversity in AML patients likely created a larger reservoir for unique resistance genes. However, EOS, antibiotics and other treatments altered the gut microbiota, reducing species diversity and thus the unique species-specific resistance genes in the milieu. Our findings are broadly consistent with observations from other immunocompromised patients, where both aerobic and anaerobic antibiotic exposures were associated with a decrease in the number of bacterial species and the number of unique ARGs within the gut metagenome of HSCT patients.^81^ Additionally, a study in pediatric leukemia patients reported that specific taxa decrease jointly with certain ARGs in response to repeated or prolonged courses of antibiotics.^82^

SMS allowed us to extend our investigation to the gut resistome, enabling the exploration of resistome associations with infection and/or colonization with AR-threat pathogens. Our findings revealed that AML patients colonized by AR-threat pathogens tended to have a greater abundance of genes related to resistance to beta-lactams and tetracycline compared to those who did not experience AR colonization (Supplemental Table 9). Although, patients with ARGs encoding aminoglycoside/quinolone resistance at baseline had ~13x greater odds of developing ARI, those with lipopeptide resistance genes had 5x greater odds of developing ARC, the large confidence intervals and lack of significance suggest that the variability in the data and that our sample size does not provide a robust representation of the population mean (Supplemental Table 10). Meanwhile, the presence of genes predicted to encode trimethoprim and aminoglycoside at baseline was significantly associated with reduced odds of ARC (Supplemental Table 3). A small cohort study involving eight children undergoing HCT for high-risk acute leukemia identified genes mediating resistance to tetracycline, macrolide, beta-lactam, and aminoglycoside resistance as the most frequently observed ARGs.^83^ A similar pattern was observed in another study on children during HSCT.^81^ Similarly, notably, the KEGG analysis indicated that the penicillin and cephalosporin biosynthesis pathways were significantly enriched in patients with AR-colonization. Moreover, a network analysis at baseline illustrated that many of the ARGs (blue) clustered together, forming a somewhat segregated region within the network. These findings indicate that these ARGs may co-occur frequently across multiple bacterial species and could represent a “shared resistance pool” accessible to various bacteria, likely through horizontal gene transfer mechanisms. These findings suggest that the baseline resistome plays a critical role in AR-outcomes.

Analysis of changes in the gut microbiome over time revealed distinct patterns between patients who experienced AR-threat events and those who did not. Patients who experienced AR colonization tended toward (*p* = 0.083) lower microbial diversity at BL. In contrast, those without AR events began with more diverse gut microbiota but experienced a significant decline in diversity in response to EOS. It is possible that patients who were eventually colonized by resistant organisms exhibited lower microbial diversity at BL, allowing opportunistic pathogens to colonize due to a lack of colonization resistance. This finding also suggests that these pathogenic bacteria might be present early on and continue to outcompete other diverse species, establishing a stable presence under selective pressure and leading to a continued decrease in diversity over time. In contrast, patients with initially diverse microbiomes, mostly composed of antibiotic-sensitive species, were more susceptible to disruption from chemotherapy and antibiotic treatment, resulting in a significant loss of microbial diversity over time.

The major limitation of our study was the small number of patients with ARI and ARC events, which reduced our ability to find statistically relevant findings. While this does not invalidate our current results, it may have influenced the precision of our estimates and our ability to detect subtle associations. However, the key advancement of this study lies in the provision of microbiome data at the species and gene levels, coupled with the incorporation of resistome data, to explore factors that influence the acquisition of medically relevant AR pathogens.

## Supplementary Material

Supplementary material Supplementary Figures and Tables.

## Data Availability

All raw data are available in publicly accessible at https://www.ncbi.nlm.nih.gov/sra. Specifically, the 16S rRNA gene sequencing data can be found under BioProject ID PRJNA1124986, while the raw data for bacterial isolates are available under BioProject ID PRJNA1129516. Additionally, shotgun metagenomic data can be accessed under BioProject IDs PRJNA1129514 and PRJNA1128111.
