# Supplementary material for: Gut microbiome features and resistome elements associated with colonization and infection with antibiotic-resistance threats
Source: Gut Microbes Rep. 2025 Oct 26;2(1):2570502. doi: 10.1080/29933935.2025.2570502 (PMC12940128; doi:10.1080/29933935.2025.2570502)
Supplement: Supplementary material — Supplementary Figures and Tables. [file KGMR_A_2570502_SM2724.zip › Supplemental material/Tables/Supplemental Table 1_121324.docx]

**Supplemental Table 1**. List of patients with ARC and their respective antimicrobial-resistant colonization isolates derived from stool.

| **Cohort** | **Patient #** | **Sample ID** | **Isolated species** | **Source** | **Marker Traits** | **Resistance to antibiotics^#^** |
| --- | --- | --- | --- | --- | --- | --- |
| PA13 | 2 | 002. S9 Ecl | *E. cloacae* | Stool | ESBL | AMX, TZP, CFZ, INN, CXM, FOX, POD, CAZ, CTR, CZX, CPM, AZT |
| PA13 | 2 | 002. S9 KP | *K. pneumoniae* | Stool | ESBL+ | AMP, AMX, TZP, CFZ, CXM, CFX, CPD, CAZ, CTR, CZX, CPM, AZT, GM, TM, CIP, LVX, MXF, TET, TGC, SXT |
| PA13 | 7 | 007. S7 KP | *K. pneumoniae* | Stool | ESBL+ | AMP, AMX, TZP, CFZ, INN, CXM, FOX, POD, CAZ, CTR, CZX, CPM, AZT, GM, TM, CIP, LVX, MXF, TET, TGC, SXT |
| PA13 | 7 | 007.S7 PA | *P. aeruginosa* | Stool | CRE+ | TZP, CFZ, CTX, CAZ, CPM, IP, MP, GT, CIP, LVX, NOR, TGC |
| PA13 | 28 | 028.S11 | *E. faecium* | Stool | Van + | AMP, SM, CIP, LVX, ERY, VAN, TET |
| PA13 | 28 | 028.S21 | *K. pneumoniae* | Stool | ESBL+ | AMP, AMX, TZP, CFZ, INN, CXM, FOX, CPD, CAZ, CTR, CZX, CPM, AZT, GM, TM, CIP, LVX, MXF, TET, TGC, NIT, SXT |
| PA13 | 45 | GAP-20 | *E. faecium* | Rectal swab | Van+ | STR, CIP, LVX, ERY, VAN, TET |
| PA13 | 78 | 078.S5 | *E. faecium* | Stool | Van + | AMP, CIP, LVX, VAN, DOX, TET |
| PA13 | 78 | GAP-51 | *E. faecium* | Rectal swab | Van+ | CIP, LVX, VAN, DOX, TET |
| PA13 | 81 | 081. S7 | *E. cloacae* | Stool | ESBL+ CRE+ | AMX, TZP, CFZ, INN, CXM, CFX, POD, CAZ, CTR, CZX, CPM, AZT, ETP, IM |
| PA15 | 2 | 002.V18 | *E. coli* | Stool | ESBL+ | TIC, PIP, INN, CXM, CEFTIN, POD, CTX, CZX, AZT, NAL, MXF, NOR, TET, AMP, AMC, SAM, CFZ, CAZ, CTX, CPM, GM, CIP, LVX, SXT |
| PA15 | 5 | 005.V2 | *S. aureus* | Stool | MRSA, Cefoxitin + | OX, CIP, LVX, ERY, CM |
| PA15 | 10 | 010.V11 | *E. coli* | Stool | ESBL+ | TIC, PIP, INN, CXM, CEFTIN, POD, CFX, CTX, CZX, AZT, NAL, MFX, NOR, TET, AMP, AMC, SAM, CFZ, CAZ, CTX, CPM, GM, CIP, LVX, SXT |
| PA15 | 10 | 010.V7 | *E. faecium* | Stool | Van+ | CIP, LVX, ERY, VAN, DOX, TET, NIT, HLG |
| PA15 | 13 | 013.V4 | *S. aureus* | Stool | MRSA, Cefoxitin + | OX, CIP, LVX, ERY, CM |
| PA15 | 13 | 013.V5 | *E. coli* | Stool | ESBL+ | TIC, PIP, INN, CXM, CEFTIN, POD, CTX, CZX, AZT, NAL, MFX, NOR, TET, AMP, AMC, SAM, CFZ, CAZ, CTX, CPM, TM, CIP, LVX, SXT |
| PA15 | 17 | 017.V3 | *S. aureus* | Stool | MRSA, Cefoxitin + | OX, CIP, LVX, ERY, CM |
| PA15 | 19 | 019.V6 | *E. faecium* | Stool | Van+ | CIP, LVX, ERY, VAN, DOX, TET, NIT, HLS |
| PA15 | 33 | 33.BL | *E. faecium* | Stool | Van+ | CIP, LVX, ERY, VAN, DOX, TET, NIT, HLS, TIC, PIP, INN, CXM, CEFTIN, CTT, CFX, POD, CTX, CTZ, AZT, MRP, AMK, NA, MFX, NOR, TET, AMP, AMC, SAM, TZP, CFZ, CAZ, CTX, CPM, ETP, TM, SXT |
| PA15 | 35 | 035.V9 | *S. aureus* | Stool | MRSA, Cefoxitin + | OX, CIP, LVX, ERY, CD, GM, TET, SXT |

# Antibiotics abbreviations are according to the Clinical and Laboratory Standards Institute (CLSI)
