# Supplementary material for: Gut microbiome features and resistome elements associated with colonization and infection with antibiotic-resistance threats
Source: Gut Microbes Rep. 2025 Oct 26;2(1):2570502. doi: 10.1080/29933935.2025.2570502 (PMC12940128; doi:10.1080/29933935.2025.2570502)
Supplement: Supplementary material — Supplementary Figures and Tables. [file KGMR_A_2570502_SM2724.zip › Supplemental material/Tables/Supplemental Table 3 120324.docx]

| **Bacterial isolates** | **Isolate ID** | **Isolate type** | **Sequence type (Sts)** |
| --- | --- | --- | --- |
| *E. coli* | MB-2822 | Infectious | 361 |
|  | MB-3009 | Infectious | 44 |
|  | MB-3021 | Infectious | 44 |
|  | MB-3042 | Infectious | 167 |
|  | MB-3567 | Infectious | 131 |
|  | MB-8751 | Infectious | 44 |
|  | 002.V18 | Colonizing | 167 |
|  | 13.V5 | Colonizing | 167 |
| *E. cloacae* | 002.S9 | Colonizing | 252 |
|  | 81.S7 | Colonizing | 732 |
| *E. faecium* | 028.S11 | Colonizing | 18 |
|  | Gap-20 | Colonizing | 1516 |
|  | 078.S5 | Colonizing | 1516 |
|  | Gap-51 | Colonizing | 665 |
|  | 028.S21 | Colonizing | 17 |
| *K. pneumoniae* | 007.S7 | Colonizing | 29 |
|  | 002.S9 | Colonizing | 29 |
|  | 028.S21 | Colonizing | 29 |
| *P. aeruginosa* | Gap-4 | Infectious | 179 |
|  | Gap-6 | Infectious | 179 |
|  | Gap-8 | Infectious | 298 |
|  | Gap-23 | Infectious | 111 |
|  | Gap-24 | Infectious | 111 |
|  | 007.S7 | Colonizing | 298 |
| *S. aureus* | Gap-5 | Infectious | 105 |
|  | 005.V2 | Colonizing | 5 |
|  | 13.V4 | Colonizing | 5 |
|  | 17.V3 | Colonizing | 105 |
|  | 35.V9 | Colonizing | 5 |

**Supplemental Table 3**. Multilocus sequence typing (MLST) to characterize infectious and colonizing isolates derived from AML patients
