# Supplementary material for: Gut microbiome features and resistome elements associated with colonization and infection with antibiotic-resistance threats
Source: Gut Microbes Rep. 2025 Oct 26;2(1):2570502. doi: 10.1080/29933935.2025.2570502 (PMC12940128; doi:10.1080/29933935.2025.2570502)
Supplement: Supplementary material — Supplementary Figures and Tables. [file KGMR_A_2570502_SM2724.zip › Supplemental material/Tables/Supplemental Table 2 _121324.docx]

**Supplemental Table 2**. List of patients with ARI and their respective antimicrobial resistant bacterial isolates derived from infections.

| **Cohort** | **Patient #** | **Sample ID** | **Isolated species** | **Source** | **Marker Traits** | **Resistance to antibiotics^#^** |
| --- | --- | --- | --- | --- | --- | --- |
| PA13 | 2 | GAP-4 | *P. aeruginosa* | Blood | CRE+ | CIP, IP, MP, TCC |
| PA13 | 2 | GAP-5 | *S. aureus* | Urine | MRSA | CM, ERM, GT, LVX, LNZ, OX |
| PA13 | 2 | Gap-6 | *P. aeruginosa* | Urine | CRE+ | CPM, CIP, IM, MRP, TCC |
| PA13 | 7 | GAP-8 | *P. aeruginosa* | Throat swab | CRE+ | AZT, CPM, CAZ, CIP, IP, MP, TZP, TCC, TM |
| PA13 | 41 | GAP-23 | *P. aeruginosa* | Blood | CRE+ | TZP, CFZ, IPM, GEN, TBM, CIP, LVX |
| PA13 | 41 | GAP-24 | *P. aeruginosa* | Bronchial Lavage | CRE+ | TZP, CFZ, IPM, GEN, TBM, CIP, LVX |
| PA15 | 2 | MB2282 | *E. coli* | Blood | ESBL+ | AMP, SAM, AZT, CTX, POD, CAZ, CTX, CIP, GEN, LVX, MXF, SXT |
| PA15 | 21 | MB3042 | *E. coli* | Blood | ESBL+ | AMP, SAM, AZT, CPM, CTX, POD, CAZ, CTX, CIP, GT, LVX, MXF, TM, SXT |
| PA15 | 26 | MB3009 | *E. coli* | Blood | ESBL+ | AMC, AMP, SAM, AZT, CTX, POD, CAZ, CTX, CIP, LVX, SXT, TM, TZP, MXF, CPM |
| PA15 | 26 | MB3021 | *E. coli* | Blood | ESBL+ | AMC, AMP, SAM, AZT, CTX, POD, CAZ, CTX, CIP, LVX, SXT, TM, TZP, MXF, CPM |
| PA15 | 29 | MB3567 | *E. coli* | Blood | ESBL+ | AMP, AZT, CTX, POD, CAZ, CTX, CIP, LVX, MXF |
| PA15 | 46 | MB8751 | *E. coli* | Blood | ESBL + | TM, CIP, CTX, CAZ, CPM, AZT, SAM |

# Antibiotic abbreviations are according to the Clinical and Laboratory Standards Institute (CLSI)
