# Supplementary figures and images for: Gut microbiome features and resistome elements associated with colonization and infection with antibiotic-resistance threats
Source: Gut Microbes Rep. 2025 Oct 26;2(1):2570502. doi: 10.1080/29933935.2025.2570502 (PMC12940128; doi:10.1080/29933935.2025.2570502)

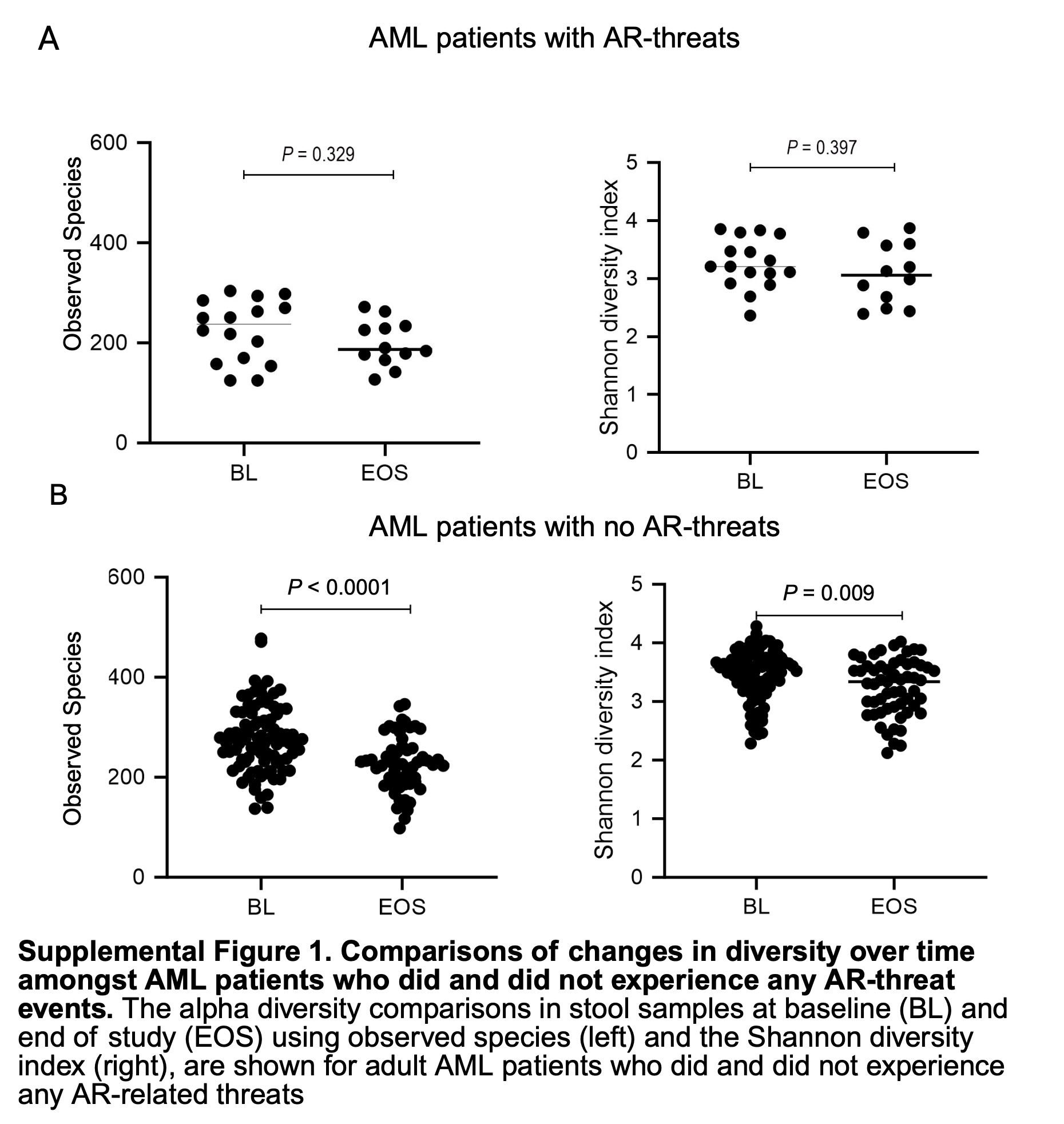

Supplement: Supplementary material — Supplementary Figures and Tables. [file KGMR_A_2570502_SM2724.zip › Supplemental material/Figures/Supplemental Figure 1.jpeg]

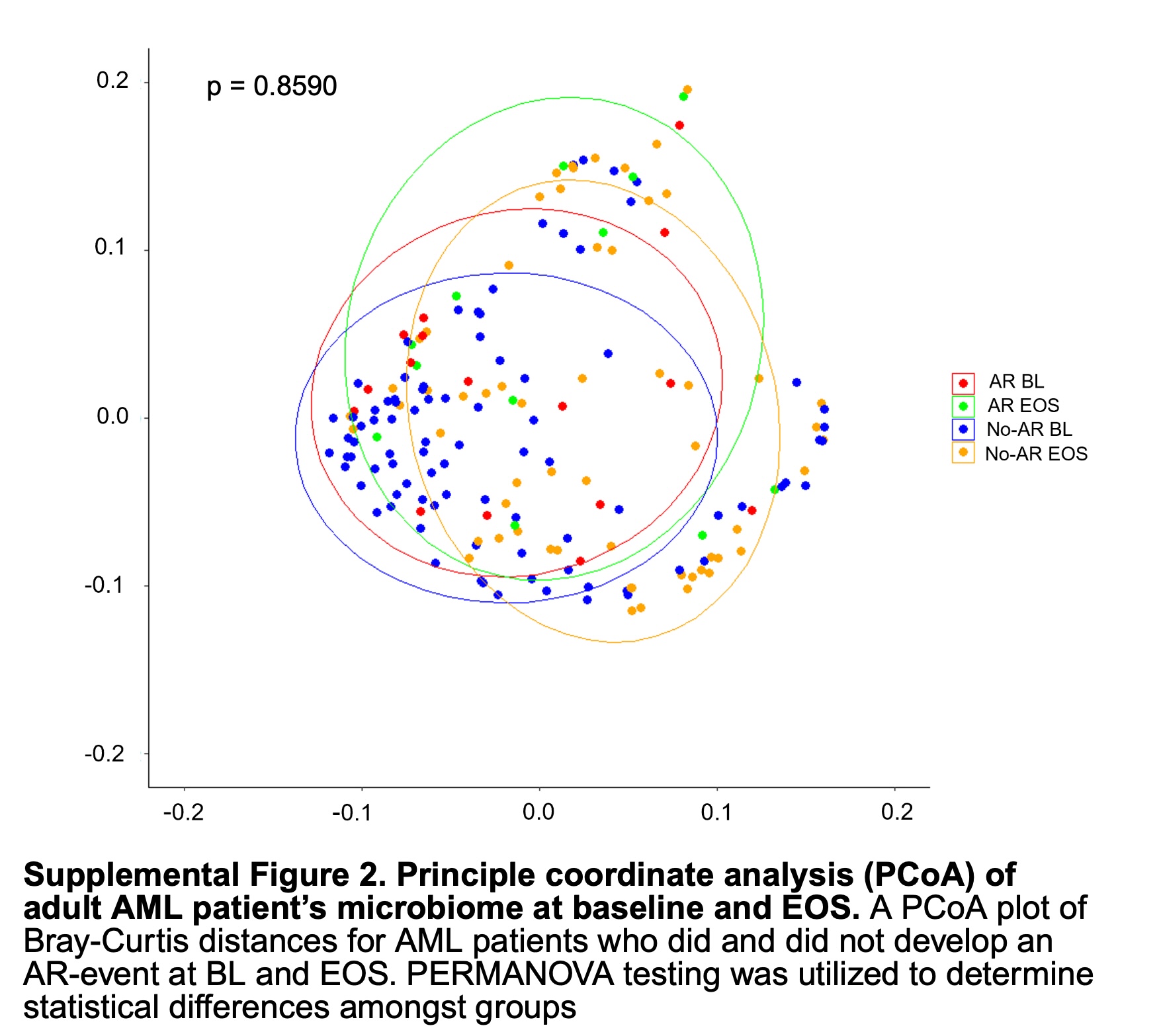

Supplement: Supplementary material — Supplementary Figures and Tables. [file KGMR_A_2570502_SM2724.zip › Supplemental material/Figures/Supplemental Figure 2.jpeg]
